# Systematic review of therapeutic outcomes of multidrug resistant tuberculosis and their predictors in adults receiving integrated treatment of tuberculosis and human immuno-deficiency virus in low- and middle-income countries: a study protocol

**DOI:** 10.1186/s13643-020-01493-5

**Published:** 2020-10-06

**Authors:** Benjamin Momo Kadia, Desmond Aroke, Kevin Pene Njefi, Joel Noutakdie Tochie, Frank-Leonel Tianyi, Reine Suzanne Kadia, Christian Akem Dimala

**Affiliations:** 1 Department of Clinical Sciences, Liverpool School of Tropical Medicine, Liverpool, UK; 2 Health Education and Research Organization (HERO), Buea, Cameroon; 3 Health and Human Development (2HD) Research Network, Douala, Cameroon; 4 Faculty of Public Health and Policy, London School of Hygiene and Tropical Medicine, London, UK; 5 Faculty of Medicine and Biomedical Sciences (FMBS), University of Yaounde I, Yaounde, Cameroon; 6 Nuffield Department of Population Health, University of Oxford, Oxford, UK; 7 Global Health Systems Solutions, Douala, Cameroon; 8 Acute Medicine Unit, University Hospitals of Leicester, Leicester, UK

**Keywords:** Integrated treatment, HIV, Multidrug-resistant tuberculosis, Outcomes

## Abstract

**Background:**

Programs that integrate tuberculosis (TB) and human immunodeficiency virus (HIV) treatment aim to provide efficient treatment services and maximize successful treatment outcomes through the delivery of both TB and HIV treatment by one provider at the same time and location. However, multi-drug resistant tuberculosis (MDR-TB) is more difficult to treat as compared to drug-sensitive TB, and in low- and middle-income countries (LMICs), the potential of programs integrating TB/HIV treatment to sustain favourable MDR-TB treatment outcomes is poorly elucidated. The objective of this review is to perform a systematic collection, critical appraisal and synthesis of existing evidence on therapeutic outcomes of MDR-TB and their predictors among adults receiving integrated treatment for TB/HIV in LMICs.

**Methods:**

A systematic review of quantitative evidence from observational cohort studies will be performed. MEDLINE, Embase, and Global Health electronic databases will be searched for relevant studies published from March 2004 to December 2019. Two investigators will independently screen the search output, review the eligible studies, and assess the quality of the eligible studies using quality assessment tools of the National Heart Lung and Blood Institute. Random-effects meta-analysis will be used to obtain summary estimates. Heterogeneity across studies will be assessed using the I^2^ statistic. The confidence in the summary estimates will be rated using the Grading of Recommendations, Assessment, Development and Evaluation (GRADE) approach. The final review will be reported following the guidelines of the Preferred Reporting System for Systematic Reviews and Meta-analysis, presented at scientific conferences and published in a peer-reviewed journal.

**Discussion:**

This study is expected to report the performance of integrated TB/HIV treatment programs as regards their potential to uphold successful MDR-TB treatment outcomes in LMICs. Furthermore, the review will indicate patient-related and healthcare-related factors that should be addressed to improve on survival of patients with MDR-TB/HIV co-infection in LMICs.

**Systematic review registration:**

This review has been registered with the International Prospective Register of Systematic Reviews and the reference ID is CRD42020159745

## Background

Tuberculosis (TB) is a leading cause of mortality worldwide and the highest burden is in low-and middle-income settings [1]. In these settings, mortality from TB is greatest among persons living with HIV/AIDS (PLWHA) [1, 2]. Some progress has been made in addressing this issue through the introduction and progressive scale-up of integrated treatment of TB and HIV [3]. The aim of the intervention is to secure efficient treatment services and maximize successful treatment outcomes for TB/HIV co-infection through the delivery of both anti-retroviral and anti-tuberculous drugs at the same time and location by one healthcare team or provider. While acknowledging the intended role of treatment integration in reducing the burden of TB in PLWHA [4, 5], the scale-up of the intervention is confronted by an important challenge: the emergence and spread of multi-drug-resistant tuberculosis (MDR-TB). This form of TB occurs when *Mycobacterium tuberculosis* cannot be killed by isoniazid and rifampin; the two best antibiotics that are most commonly used to treat TB [6, 7]. MDR-TB primarily results from mismanagement of treatment (as in poor treatment adherence and use of low-quality drugs) and person-to-person transmission of the resistant strains which ultimately persist in the population [7]. The survival and spread of the resistant strains are potentiated by a global lack of appropriate measures to detect and treat them promptly [8]. Overall, MDR-TB is more complicated and expensive to treat than the ordinary drug-sensitive TB [9]. It remains an important barrier to successful TB treatment and in 2018, 500,000 new cases of MDR-TB were recorded worldwide [1].

While novel strategies are being designed and investments made to scale-up the delivery of integrated treatment for TB/HIV, there remains a huge knowledge gap on treatment outcomes of MDR-TB within programs integrating TB/HIV treatment in low- and middle-income settings where the burden of TB/HIV co-infection is highest. Previous studies have generally focused on the potential of integrated TB/HIV treatment programs to sustain successful TB treatment outcomes in patients co-infected with HIV and drug-sensitive tuberculosis. A recent systematic review by Chem et al. investigated treatment outcomes in patients co-infected with MDR-TB and HIV in sub-Saharan Africa (SSA) and found that whilst MDR-TB treatment outcomes are comparable to those reported globally, successful outcomes are much lower in patients who are co-infected with HIV [10]. Notwithstanding these pertinent findings, the review was limited to SSA [10] whilst recent global reports indicate relatively higher rates of TB in general and MDR-TB in particular in low-income Asian settings compared to SSA [11], albeit these observations may be due to under-diagnosis or under-reporting which remain serious problems in SSA. Furthermore, an important objective of integrating TB/HIV treatment programs is to start anti-TB drugs early for PLWHA who are diagnosed with TB, with the intent of limiting progression to more severe TB and improve survival. However, a recent systematic review by Harris et al. noted that evidence supporting the superiority in the effectiveness of early initiation of treatment of MDR-TB over that of delayed treatment is insufficient [12]. Evidence on other factors that could predict MDR-TB treatment outcomes in the general population and in PLWHA more specifically, are also poorly understood. Broader perspectives into the potential of TB/HIV integrated treatment services in LMICs to uphold successful MDR-TB outcomes can be derived by synthesising evidence on both MDR-TB treatment outcomes and predictors of these outcomes when TB/HIV services are integrated in LMICs. These will help formulate strategies to better equip integrated treatment programs for the effective management of MDR-TB/HIV co-infection and increase MDR-TB treatment success.

## Objectives

1. To synthesise quantitative estimates of treatment outcomes of MDR-TB in adults receiving integrated treatment for TB and HIV in LMICs.
2. To determine the predictors of MDR-TB treatment outcomes in this population.

## Methods

In order to attain our objectives, the following steps will be followed:

1. Development of a literature search strategy to identify evidence on MDR-TB treatment outcomes among adults receiving integrated therapy for TB/HIV in LMICs.
2. Screening of all the identified studies in objective 1 for their relevance in addressing the research objectives.
3. Critical appraisal of the evidence obtained from studies retained from objective 2
4. Extraction of relevant data from studies in [3] on treatment outcomes of MDR-TB and their predictors in adults receiving integrated treatment for TB/HIV in LMICs.
5. Meta-analysis (if justified) of the evidence obtained in [4].

### Search strategy

MEDLINE, Embase, Global Health, and Cochrane electronic databases will be searched extensively to include studies published between March 2004 (when the World Health Organization issued the first guidelines on TB and HIV collaborative activities) and December 2019. A data extraction form and definition of key terms will be developed to standardise the data collection process. The search terms and their variations will be used in combination as shown on Table 1. Articles retrieved from the search will be saved on Mendeley desktop software and their titles and abstracts (including those in reference lists of relevant articles) will be screened. Following the screening, studies that fulfil the inclusion criteria and adequately address the research objectives shall be retained for full-text review. The reference lists of included studies and previous reviews will be explored to identify other eligible studies.

**Table 1:** Search strategy for systematic review

Search #	Search query
1	Integrat* OR joint OR collaborat* OR concurrent OR concomitant
2	Treat* OR therap* OR care OR service OR manag*
3	‘Human immun?deficiency virus’ OR HIV OR HIV1 OR HIV2 OR AIDS OR ‘Acquired immune?deficiency syndrome’ OR anti?retroviral
4	Tuberculosis OR ‘Mycobacterium tuberculosis’ OR ‘Pulmonary tuberculosis’ OR TB OR anti?tubercul*
5	#1 AND #2 AND #3 AND #4

### Selection criteria

The study will include peer-reviewed quantitative and mixed methods studies describing MDR-TB treatment outcomes and their predictors in the context of integrated TB/HIV treatment in LMICs. These studies will include observational cohort and case control studies published in the English language. BMK and DA will independently assess retrieved titles and abstracts of relevant studies for their eligibility to be included in the review. They will then perform independent screening of the full texts of retained articles. Conference abstracts, editorials, letters to the editor, bulletins, and grey literature shall be excluded. Disagreements between the two investigators will be resolved by arbitration by a third investigator (CAD). Table 2 shows the inclusion and exclusion criteria set for the study. These criteria were defined using the PICOS (Population, Intervention, Comparator, Outcome, Study design) approach.

**Table 2:** Selection criteria for studies to be included in systematic review of therapeutic outcomes of multi-drug-resistant tuberculosis and their predictors in adults receiving integrated treatment of tuberculosis and human immuno-deficiency virus in low- and middle-income countries

Item	Inclusion criteria	Exclusion criteria
Population	Studies involving adults who are co-infected with MDR-TB and HIV in LMICs	Studies involving -Pregnant women -Patients with multi-morbidities
Intervention	Studies reporting on the delivery of integrated treatment of TB and HIV in LMICs	-Studies describing treatment of only MDR-TB -Studies describing integrated treatment of drug-sensitive TB and HIV -Studies describing integrated treatment of more than 2 diseases (TB/HIV)
Comparison		
Outcome(s)	Studies reporting on MDR-TB outcomes and/or their predictors in the setting of integrated therapy for TB/HIV.	Studies describing outcomes that are unrelated to treatment of MDR-TB in the context of integrated TB/HIV therapy
Study design	Observational studies specifically, cohort studies that use quantitative research methods	1) Interventional studies 2) Observational studies other than cohort, e.g. cross-sectional studies 3) Mini-reviews, conference abstracts, letters to editors, editorials, commentaries. 4) Studies whose full data will not be available even upon requesting from the author 5) Duplicates studies: for studies published with the same or different titles or in more than one journal, the most updated version shall be considered.

### Data extraction and analysis

Relevant data will be extracted from each eligible article retained for full-text review. The extracted data will be saved on a Microsoft Excel 2016 form and subsequently double-checked for accuracy by the investigators. The data to be extracted include the following:

1. Publication details: name of first author, publication year, journal reference, country, and place of study, year of study, study design, study area and setting, study population, sample size, characteristics of study participants (such as age and sex distribution), as well as limitations and strengths of studies.
2. Outcomes of interest: Treatment outcomes of multi-drug-resistant pulmonary TB as defined by the World Health Organisation (WHO): cured, treatment completed, defaulted, and mortality [12, 13]. Multi-drug-resistant TB is considered when *Mycobacterium tuberculosis* cannot be killed by at least isoniazid and rifampin [6, 7, 10]. Cure is defined as the presence of a negative sputum smear at the last month of treatment and at least on one other occasion during treatment. Treatment completion is the term used to describe a patient who completed treatment, but for whom smear examination results were not complete enough to classify the patient as cured; or based on clinical, radiological and complementary examination criteria in those patients who did not produce sputum for a smear examination. Death refers to all-cause mortality occurring after TB diagnosis and before the end of treatment. A defaulter is one who fails to collect medicines for ≥ 2 consecutive months. According to the guidelines, successful treatment outcomes refer to the combination of ‘cure’ and ‘treatment completed’ categories. As per the WHO classification, successful treatment refers to cases of treatment completion and cure [12, 13]. Measures of frequency and central tendency referring to these outcom,es will be recorded as well. Predictors of treatment outcomes will be considered as all variables which studies will report as having an association with MDR-TB treatment outcomes. Data on predictors of each of the WHO TB treatment outcomes will be extracted.

BMK and DA will independently assess the quality of quantitative evidence from quantitative and mixed methods studies using appropriate tools in the National Heart, Lung and Blood Institute (NHLBI). Disagreements in the quality of studies will be resolved by arbitration by a third investigator (CAD). Data extracted from the retained studies will be recorded on Microsoft Excel 2016 and quantitative data exported to STATA 15. Random-effects meta-analysis will be used to analyse and synthesise data on treatment outcomes. For predictors of treatment outcomes, random-effects meta-analysis will be performed to obtain pooled effect estimates summarizing data on predictors of each of the four treatment outcomes, i.e. cure, treatment completion, defaulted, and death. Any test statistic that can be converted to effect size will be considered but we anticipate that correlation and regression coefficients will be the most reported measure of effect and will therefore be the effect sizes of primary interest in the review. For studies that report odds ratios, the log odds will be calculated prior to pooling the effect sizes. In order to explore the possible effects of confounding, separate analyses will be performed for adjusted and unadjusted effect estimates. When methodological limitations could potentially explain the effect size in a study, sensitivity analysis will be conducted by omitting the study and repeating the meta-analysis. Data on treatment outcomes and effect of predictors will be represented on forest plots and risk of bias will be assessed using funnel plots. The degree of variability in this data will be assessed by visual inspection of the forest plots and interpretation of the *I*^2^ statistic from meta-analyses. Based on the *I*^2^ statistic, the degree of variability or heterogeneity will be interpreted as none (*I*^2^ < 25%), low (25 ≤ *I*^2^ ≤ 49%), moderate (50 ≤ *I*^2^ ≤ 74%), or high (*I*^2^ ≥ 75%). For each treatment outcome, the p-value for heterogeneity shall be used to determine whether the heterogeneity is associated with variations in the observed effect size across studies. In case of variability *I*^2^ > 50% in the effect of predictors, the latter will be synthesised using a qualitative approach. Where possible, meta-regression will be used to assess the effect of variations in sample size, study design, and geographical location on summary estimates. All reported *p* values will be two-sided with a significance level of 0.05. The confidence in the summary estimates will be rated using criteria of the Grading of Recommendations, Assessment, Development and Evaluation (GRADE) approach [14].

### Reporting

This protocol has been reported in accordance with the Preferred Reporting Items for Systematic Reviews and Meta-Analysis Protocol (PRISMA-P) [15, 16] (supplementary file 1).

### Systematic review registration

This review has been registered with the International Prospective Register of Systematic Reviews and the reference ID is CRD42020159745.

## Discussion

There is paucity of evidence on therapeutic outcomes of MDR-TB/HIV co-infection when managed within TB/HIV integrated treatment programs in LMICs. This review will involve a systematic collection, critical appraisal and synthesis of evidence on therapeutic outcomes of MDR-TB among adults receiving integrated treatment for TB/HIV in LMICs. This study will report the performance of integrated TB/HIV treatment programs in LMICs by describing the potential of these programs to uphold successful outcomes among MDR-TB/HIV co-infected patients. Furthermore, the study will discuss factors that should be addressed to improve on survival of patients with MDR-TB/HIV co-infection who receive integrated treatment in LMICs. The final review will be presented at scientific conferences and published in a peer-reviewed journal.

## Supplementary information

**Additional file 1.** PRISMA-P 2015 checklist for protocol on systematic review of therapeutic outcomes of MDR-TB and their predictors in adults receiving integrated treatment of tuberculosis and Human Immuno-deficiency Virus in low and middle-income countries.

## Data Availability

Not applicable

## Abbreviations

HIV: Human immunodeficiency virus
AIDS: Acquired immunodeficiency syndrome
LMICs: Low- and middle-income countries
MDR: Multi-drug resistant
PLWHA: Persons living with HIV/AIDS
TB: Tuberculosis
